# Reduction of *Salmonella* contamination on the surface of chicken skin using bacteriophage

**DOI:** 10.1186/s12985-020-01368-0

**Published:** 2020-07-09

**Authors:** Robert Joseph Atterbury, Adriano Marcelo Gigante, María de la Salud Rubio Lozano, Ruben Danilo Méndez Medina, Gareth Robinson, Habib Alloush, Paul Andrew Barrow, Vivien Mary Allen

**Affiliations:** 1 Department of Clinical Veterinary Science, University of Bristol, Langford, UK; 2 Present Address: School of Veterinary Medicine and Science, University of Nottingham, Sutton Bonington, Leicestershire LE12 5RD UK; 3 Facultad de Medicina Veterinaria y Zootecnia, Universidad Nacional Autonoma de Mexico, Mexico City, Mexico; 4 Faculty of Health and Applied Sciences, University of the West of England, Bristol, UK

**Keywords:** Bacteriophage, *Salmonella*, Biocontrol, Chicken, Skin

## Abstract

**Background:**

Enteric infections caused by *Salmonella* spp. remain a major public health burden worldwide. Chickens are known to be a major reservoir for this zoonotic pathogen. The presence of *Salmonella* in poultry farms and abattoirs is associated with financial costs of treatment and a serious risk to human health. The use of bacteriophages as a biocontrol is one possible intervention by which *Salmonella* colonization of chickens could be reduced. In a prior study, phages Eϕ151 and Tϕ7 significantly reduced broiler chicken caecal colonization by *S.* Enteritidis and *S.* Typhimurium respectively.

**Methods:**

*Salmonella*-free Ross broiler chickens were orally infected with *S.* Enteritidis P125109 or *S.* Typhimurium 4/74. After 7 days of infection, the animals were euthanased, and 25cm^2^ sections of skin were collected. The skin samples were sprayed with a phage suspension of either Eϕ151 (*S.* Enteritidis), Tϕ7 phage suspension (*S.* Typhimurium) or SM buffer (Control). After incubation, the number of surviving *Salmonella*s was determined by direct plating and Most Probable Number (MPN). To determine the rate of reduction of *Salmonella* numbers on the skin surface, a bioluminescent *S*. Typhimurium DT104 strain was cultured, spread on sections of chicken breast skin, and after spraying with a Tϕ11 phage suspension, skin samples were monitored using photon counting for up to 24 h.

**Results:**

The median levels of *Salmonella* reduction following phage treatment were 1.38 log_10_ MPN (Enteritidis) and 1.83 log_10_ MPN (Typhimurium) per skin section. Treatment reductions were significant when compared with *Salmonella* recovery from control skin sections treated with buffer (*p* < 0.0001). Additionally, significant reduction in light intensity was observed within 1 min of phage Tϕ11 spraying onto the skin contaminated with a bioluminescent *Salmonella* recombinant strain, compared with buffer-treated controls (*p* < 0.01), implying that some lysis of *Salmonella* was occurring on the skin surface.

**Conclusions:**

The results of this study suggest that phages may be used on the surface of chicken skin as biocontrol agents against *Salmonella* infected broiler chicken carcasses. The rate of bioluminescence reduction shown by the recombinant *Salmonella* strain used supported the hypothesis that at least some of the reduction observed was due to lysis occurred on the skin surface.

## Background

Salmonellosis is one of the most commonly reported food borne diseases worldwide and remains a costly public health burden in many countries [1]. The World Health Organization estimates show *Salmonella* as a frequent cause of foodborne illness worldwide; responsible for 7.6 million cases in 2010, being *Salmonella enterica* accounted for 59, 000 and *Salmonella* Typhi for 52, 000 deaths [1].

Over 45, 000 cases of human salmonellosis were reported in the US in 2016 [2], and over 90, 000 cases reported in the EU in 2017 [3]. In 2013, the United States Department of Agriculture estimated the total cost of human salmonellosis in the US at over $3,6 billion [4]. The most recent FoodNet 2015 annual report shows that the number of hospitalized patients due to a *Salmonella* infection was almost twice that of *Campylobacter*, and comparable to all the hospitalizations due to infections caused by *Campylobacter*, *Listeria*, *Shigella*, *E. coli*, *Vibrio* or *Yersinia* combined [5].

Poultry, and particularly chickens, are widely accepted as a major source of *Salmonella* entering the human food chain [6, 7]. Improved cleaning and disinfection, biosecurity and the use of vaccines for breeding and laying flocks have helped to reduce the prevalence of *Salmonella* in chickens [8]. However, comprehensive biosecurity on farms is expensive and difficult to maintain and is a viable option only when there is a high value product and the consequences of *Salmonella* transmission are severe [9]. Even if good biosecurity is maintained on the farm, broiler chickens may become colonised with *Salmonella* if they are transported to the abattoir in contaminated crates [10].

Approximately 13.1% of chicken carcasses sampled in the EU tested positive for *Salmonella* [11]. The options for reducing contamination at this stage are limited by EU legislation. In the United States, contaminated broiler chicken carcasses can be washed in water containing chlorine. However, *Salmonella* is known to attach firmly to the skin of broiler chickens [12] and may not be readily accessible to free chlorine, or be relatively unaffected by it [13, 14]. Other chemical treatments e.g. trisodium phosphate, organic acids, sodium hydroxide, sodium metabisulphite and hydrogen peroxide have been used to reduce *Salmonella* counts on chicken skin, typically by 1–2 log_10_ CFU [15]. However, some studies suggest that the concentrations of these chemicals required to significantly reduce *Salmonella* contamination can result in an unacceptable deterioration in the organoleptic quality of the treated carcasses [15]. In the EU, regulation 853/2004 provides that no substance other than water (either potable or clean) can be used to remove surface contamination from foods of animal origin. This being the case, other means of controlling *Salmonella* in poultry processing are needed [10].

Bacteriophage (phage) therapy is one method of reducing microbial contamination which has gained prominence over the years [16, 17]. Phages are natural parasites of bacteria and are ubiquitous in the environment at estimated levels of 10^30^ to 10^32^ PFU in the biome [18, 19]. The use of host-specific phage has been promoted as a cost-effective and adaptable approach to control zoonotic bacteria [20, 21]. Phages have unique advantages when compared with antibiotics [21]. For example, they replicate only in a targeted subset of bacteria, avoiding the imbalance of commensal flora (dysbiosis) often caused by the use of broad-spectrum antibiotics. Additionally, they will only replicate as long as the targeted bacterium is present and so are naturally self-limiting [22]. Phages have been used to reduce the numbers of *Campylobacter jejuni* in commercial broilers by up to 5.0 log_10_ CFU g^− 1^ caecal contents [23]. Significant reductions in *Salmonella* numbers colonizing broiler chickens by using phage have also been reported [24, 25]. Both *Campylobacter* and *Salmonella* phages can be isolated readily from poultry excreta and the poultry farm environment [26–28] and as such would not introduce any new biological entity into the food chain if used therapeutically in poultry production.

The emergence of bacteriophage insensitive mutants (BIMs) has long been perceived as a major limitation of phage therapy [22]. Unlike chemotherapeutic agents such as antibiotics, phage constantly evolve to circumvent their host’s defences and resistant bacteria are often less fit or less virulent than their phage-sensitive counterparts [29]. However, the recolonization of animals with BIMs following phage treatment has been reported for several genera of zoonotic pathogens [23, 24, 30]. Ideally, phage should be applied in such a way as to restrict the opportunities for the emergence and spread of BIMs into the environment. In situations where phage greatly outnumber their hosts, the adsorption of many phage onto a single cell can cause death by “lysis from without” [31]. If this is carried out at the end of the processing line quickly followed by refrigeration, conceptually the emergence of BIMs would be greatly limited. Phage treatment in this instance would be arguably more easily optimised and controlled than phage therapy of live animals on a farm. A washed carcass surface, whilst providing some protection to resident bacteria [32, 33], is not the viscous matrix containing many potential decoys which may be encountered in the intestinal lumen of animals [34]. As such, phage treatment of carcasses may be more efficient than medicating live animals on the farm. Here we describe the use of *Salmonella* phages to reduce the numbers of two *Salmonella* serovars (Enteritidis and Typhimurium) on the surface of broiler chicken skin. We also use a bioluminescent *Salmonella* Typhimurium DT104 strain to determine how quickly this reduction may take place on the skin surface.

## Methods

### Sources and preparation of phage

All of the phages used in this study (Eϕ151, Tϕ10 and Tϕ11) were isolated from poultry excreta and abattoir effluent as reported previously [24]. All of these phage exhibited a clear plaquing phenotype on their respective host strains of *Salmonella* used in this study, indicating a lytic lifecycle on these strains. A lytic spectrum for phage Tϕ11 is presented in Supplementary Table 1, and electron micrographs of this phage are presented in Supplementary Figures 1 and 2. Phages were propagated on their host strains in nutrient broth (NB, CM0001, Oxoid, Basingstoke, UK) using a modification of a previously described protocol [35]. Briefly, a volume of fresh overnight culture of the host strain (0.1 ml) was added to 10 ml of pre-warmed NB (37 °C) in a 30 ml tube. To this was added 0.1 ml of a 10^6^ PFU ml^− 1^ suspension of phage. The suspension was incubated statically at 37 °C for up to 8 h until lysis was apparent (compared with an uninfected control culture). The lysate was then filtered through a 0.22 μm pore-size filter (Millipore) and the phage titer determined using the method described below. Phage stocks were stored at 4 °C until required, but for no longer than 48 h before application. Phages in crude liquid lysates were concentrated and purified using PEG precipitation, as described previously [36]. Phage titres were determined by decimally diluting the phage suspension in SM buffer (50 mM Tris-Cl [pH 7.5], 0.1 M NaCl, 8 mM MgSO_4_.7H_2_O, 0.01% w/v gelatin, reagents from Sigma) then adding duplicate 100 μl volumes of each dilution to equal volumes of approximately 8.0 log_10_ CFU ml^− 1^ of an overnight NB culture of *Salmonella* host strain and incubating for 15 min at 37 °C. Each of these suspensions was then added to 5 ml of molten overlay agar (NB containing 0.5% w/v bacteriological agar LP0011, Oxoid), gently shaken, and poured over pre-warmed (37 °C, 30 min) nutrient agar plates (NA, CM0003, Oxoid). After allowing the overlay to set, the plates were incubated at 37 °C for 24 h before examining for plaques.

### Experimental birds

*Salmonella*-free Ross broiler chickens (36 days old, *n* = 36) were obtained from a commercial supplier (Lloyd Maunder, Devon, United Kingdom). Upon arrival at the University, the birds were separated into two equal groups and housed in groups of three in floor boxes in a controlled environment under strict conditions of biosecurity. To ensure that the experimental birds remained free of naturally-occurring *Salmonella* infection, faeces were collected periodically and screened for *Salmonella* by an enrichment step in modified Rappaport-Vassiliadis Soya peptone broth (RVS, CM0669, Oxoid), followed by streaking onto Brilliant Green Agar (BG, CM0329, Oxoid). Faecal (and later, caecal) samples were also collected to determine if any pre-existing *Salmonella* phages were present using the enrichment method for the environmental samples described previously [24]. On the day of arrival the birds were orally inoculated with 0.3 ml of an 8.0 log_10_ CFU ml^− 1^ suspension of either *S.* Enteritidis P125109 (group 1, *n* = 18), or *S.* Typhimurium 4/74 (group 2, *n* = 18). Both of these *Salmonella* strains were resistant to sodium nalidixate (20 μg ml^− 1^). All of the birds were killed by cervical dislocation 7 days after *Salmonella* challenge. The birds were hand plucked (without scalding or washing) and then two sections of skin (each 25 cm^2^) were carefully excised from the breast skin using sterile scissors and a disposable template, and placed into separate sterile Petri dishes. After the skin sections were collected from the birds and removed to a separate laboratory, the abdominal cavity was opened and the caeca were removed. This procedure was followed to ensure that there would be no possibility of gut contents contaminating the surface of the skin. The contents of the caecal lumen were collected in sterile universal tubes for *Salmonella* and phage enumeration using the same methods described above.

### Phage treatment of skin

A total of 36 birds were used in the experiment, half of which were orally infected with *S.* Enteritidis and half with *S.* Typhimurium (see above). Two sections of skin were collected from each bird (72 skin samples total). One section of skin from each bird was sprayed with 1 ml (0.5 ml per side) of SM buffer (control) using a hand-operated plant spray, with the nozzle positioned approximately 10 cm from the surface of the skin and delivering 0.5 ml of liquid per spray. The other section was sprayed in an identical manner with 1 ml of a 9.0 log_10_ PFU ml^− 1^ of phage Eϕ151 (group 1), or Tϕ10 (group 2) for birds challenged with *S.* Enteritidis or *S.* Typhimurium respectively. After allowing 20 min to dry in a Class II biological safety hood, each skin section was transferred into a stomacher bag containing 50 ml of maximum recovery diluent (MRD, CM0733, Oxoid) and stomached for 1 min. *Salmonella*s in the stomachate were enumerated using two parallel methods. For the first method, decimal dilutions of the stomachate were prepared in MRD. Volumes (100 μl) of each dilution were then spread-plated onto BG agar containing 25 μg ml^− 1^ sodium nalidixate (BG nal) and incubated at 37 °C for 24 h before examining for typical *Salmonella* colonies. The second method used the Most Probable Number technique (MPN) [37] using Rappaport Vassiliadis broth as the enrichment medium and BG nal agar for the plating medium.

### Determining the rate of lysis in situ

In order to determine the rate of reduction of *Salmonella* numbers on the skin surface, a series of experiments were performed using a bioluminescent *Salmonella* host strain. The strain used was *S*. Typhimurium DT104 transformed with the pBBR1MCS5-LITE lux plasmid as described previously [38]. A linear relationship between photon and viable counts has previously been demonstrated for the plasmid construct when expressed in *Pseudomonas aeruginosa* (*r*2 = 0.982) [39] and in the *S*. Typhimurium DT104 used in this study (*r*2 = 0.9856) [40]. *S*. Typhimurium DT104 pBBR1MCS-5-LITE has been used successfully to assess the impact of in situ rapid heating and cooling on food surfaces, showing a strong correlation between bioluminescence and cell numbers (*r*^2^ = 0.97) [41, 42].

Sections of chicken breast skin were inoculated with 100 μl of a 10^6^ CFU ml^− 1^ suspension of an overnight NB culture of the bioluminescent *Salmonella* strain, which had been washed twice and resuspended in MRD. The inoculum was spread evenly over the surface of the skin and then left to dry at 20 °C for 1 h in a Class II biological safety hood. After this time, two 25 cm^2^ sections were excised from the same piece of skin for each trial. The first section of skin (control) was sprayed with 0.5 ml of MRD; the second skin section (treated) was sprayed with 0.5 ml of a 10^9^ PFU ml^− 1^ suspension of phage Tϕ11. The skin surfaces were viewed within 1 min of spraying, in a dark room with an ICCD 225 photon counting camera (Photek Ltd., St Lenards-on-Sea, East Sussex, TN38 9NS, UK). Photons were counted for 1 min at set intervals for up to 24 h at 20 °C. Each trial was repeated five times.

### Statistical treatment of data

All statistical tests were performed on log_10_ transformed data. The D’Agostino & Pearson omnibus normality test was used to determine if the data had an underlying normal distribution as this is a prerequisite for the use of parametric statistical tests. Where a normal distribution could not be determined, the median counts of *Salmonella* recovered from control and phage-treated skin sections were compared using the non-parametric Mann-Whitney U test. The proportion of control and phage-treated skin sections that were culture positive for *Salmonella* were compared using Fisher’s Exact Test. All statistical calculations were performed using SPSS® 14.0 for Microsoft Windows, SPSS Inc., Chicago, USA.

## Results

### Phage treatment of chicken skin

Data showing the recovery of *S.* Enteritidis and *S.* Typhimurium from chicken skin following treatment with phage is presented in Table 1. *Salmonella* and their phage were not recovered from any of the faecal samples taken from birds prior to experimental inoculation. No *Salmonella* phage could be isolated from the caecal content samples taken from the birds of the control group after slaughter. However, *Salmonella* spp. was recovered from the caeca of all birds used for the skin disinfection trials. The mean level of colonisation for birds infected with *S.* Enteritidis and *S.* Typhimurium (as determined from colony counts) was 4.0 ± 1.3 and 3.5 ± 1.0 log_10_ CFU g^− 1^ caecal contents respectively. When recovering *Salmonella* from some of the skin sections, all of the tubes inoculated for the MPN enumeration method tested positive for *Salmonella*. These samples were given a value of ≥3.04 log_10_ MPN per skin section (the upper detection limit of the MPN method used here). In addition, as the exact number of *Salmonella* in these samples was unknown, the mean and standard deviation could not be calculated accurately. As such, the median values and range are presented here. All of the control skin sections treated with buffer tested positive for *Salmonella* using the MPN method. The phage treatment of *S.* Enteritidis-contaminated skin sections resulted in a significant (*p* < 0.0001, Mann-Whitney U Test) median reduction of 1.38 log_10_ MPN per skin section compared with the control. Moreover, *S.* Enteritidis could not be recovered from 13/18 of the phage-treated skin sections, which was also significantly lower than the control group (*p* < 0.0001, Fisher’s Exact Test). Phage treatment of *S.* Typhimurium-contaminated skin sections led to similar results. The median recovery of *S.* Typhimurium was reduced significantly (*p* < 0.0001) by 1.83 log_10_ MPN per skin section compared with the control group. The proportion of *Salmonella*-positive skin sections following phage treatment (11/18, 61.1%) was also significantly lower than the control group (*p* = 0.0076).

**Table 1:** The recovery of *Salmonella* Enteritidis and *Salmonella* Typhimurium from the skin of experimentally-infected chickens which have been treated with a buffer (control) or phage suspension. The number of skin sections from which *Salmonella* could be recovered by MPN enrichment is given out of a total of 18. The median log_10_ MPN recovery per skin section is also given, along with the range. Skin sections which contained the same or more than the maximum detection limit for the MPN technique were assigned the value “≥ 3.04”

Bacterial contaminant	Control (buffer-treated) group		Phage-treated group	
	No. of *Salmonella* positive skin sections (%)	Median MPN per skin section (range)	No. of *Salmonella* positive skin sections (%)	Median MPN per skin section (range)
*S.* Enteritidis	18/18 (100.0)	1.38 (0.95 to ≥3.04)	5/18 (27.8)	0.00 (0.00 to 1.11)
*S.* Typhimurium	18/18 (100.0)	2.43 (1.46 to ≥3.04)	11/18 (61.1)	0.60 (0.00 to 2.66)

### Demonstration of lysis on the skin surface

**Fig. 1:**
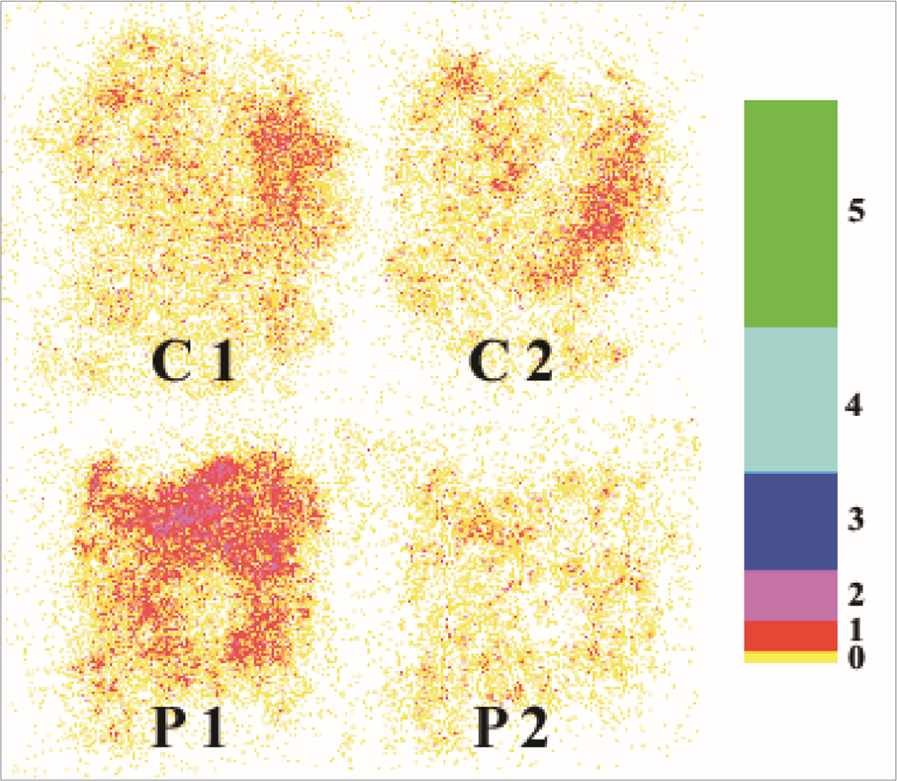
Negative image of control (C) and phage-treated (P) skin sections before spray treatment (1) and 2 min after spray treatment (2). A negative image of the logarithmic colour palette used by the Photek IFS32 software is presented on the right of the figure. Each colour represents the log_10_ number of photons collected at a given time point (1 min). The numbers on the right represent powers of ten (e.g. 2 = 10^2^ photons). All images were processed identically using Adobe® Photoshop 7.0 (Adobe Systems Inc., San Jose, USA) on a Viglen Genie XL280 PC running Microsoft Windows XP

Photographs showing the photon counts from chicken skin sections experimentally inoculated with a bioluminescent strain of *S.* Typhimurium before and after spray treatment with a buffer or phage suspension are presented in Fig. 1. The mean photon count from the skin sections before buffer or phage treatment was 4.3 log_10_ RLU per skin section. The photon count for the skin treated with buffer increased slightly to 4.4 log_10_ RLU per skin section after spraying but this increase was not significant (*p* > 0.1). The mean photon count from the skin sections treated with phage fell from 4.3 to 4.1 log_10_ RLU which was a modest but statistically significant reduction compared with the control skin sections (*p* < 0.01). Following incubation for 24 h at room temperature (20 °C), the mean photon counts increased for the control skin sections (6.1 log_10_ RLU). The mean photon counts also increased for the phage-treated skin sections but reached a level significantly lower (*p* < 0.05) than the control skin sections (5.2 log_10_ RLU).

## Discussion

The use of phages to control bacterial contamination of foods is an area of continuing interest and development. Since the FDA approved the limited use of phage to control *Listeria* in fresh foods, commercial interest in this area has increased considerably [43]. Several studies from around the world have highlighted the potential benefits of using phage to control zoonotic pathogens and food spoilage bacteria [44–47]. *Salmonella* continues to be a major global public health problem and many cases of human salmonellosis can be linked to the consumption of contaminated poultry products. It is widely accepted that poultry and associated products are a major source of entry into the human food chain for this pathogen. The entry of live birds contaminated with *Salmonella* into the abattoir can lead to widespread dissemination of this pathogen along the processing line and may lead to contamination of the final product [48]. Moreover, commercial cleaning procedures may not be sufficient to remove all *Salmonella*s from the processing line [49].

The numbers of both *S.* Enteritidis and *S.* Typhimurium were significantly reduced following phage treatment compared with the controls (*p* < 0.0001). Over 70% of the *S.* Enteritidis contaminated sections were culture-negative for *Salmonella* following phage treatment which suggests that this approach could be used in poultry processing plants to reduce the numbers of this zoonotic pathogen in the human food chain. If the chickens that arrive at the abattoir are carrying *Salmonella*, they will almost inevitably produce contaminated carcasses. In the present study, the use of commercial chickens experimentally colonised with *Salmonella* would be expected to result in a more realistic distribution of the pathogen across the carcass surface than, for example, sections of skin inoculated with a suspension of bacteria, which are models which have been used in previous studies [50, 51].

In the present study, median reductions of between 1.38 and 1.83 log_10_ MPN were achieved following phage treatment for skin contaminated with *S.* Enteritidis and *S.* Typhimurium respectively. These reductions largely agree with those recorded previously [44, 52, 53], and are similar to, or greater than reductions (~ 0.81 log_10_ CFU cm^− 2^) obtained following the chemical treatment of chicken skin with agents such as peroxyacetic acid, lactic acid and dichloroisocyanurate [50]. Indeed, this last study found that the reductions in *Salmonella* following phage treatment were not significantly different from those obtained using conventional chemical treatments at commercial levels of application. Another study found that reductions in various *Salmonella* serotypes of up to 5.0 log_10_ CFU ml^− 1^ could be achieved in vitro by combining phage and chemical treatments such as cetylpyridinium chloride and lauric arginate [53]. However, these reductions fell to between 1.6 and 2.5 log_10_ CFU cm^− 2^ when applied to chicken skin and meat surfaces.

A previous study concerning a Quantitative Risk Assessment (QRA) model of the link between the level of *Salmonella* contamination on broiler chicken carcasses and human infection indicated that a 50% reduction in chicken breast contamination at retail is predicted to reduce the probability of human infection by 40% [54]. Interestingly, the contamination levels used in this model ranged from ≤0.3 to 100 MPN g^− 1^ which is similar to levels recorded in the present study for naturally-contaminated chicken skin. This may suggest our findings are more directly relevant to existing models of *Salmonella* in the poultry meat supply chain and could be incorporated into future models examining the potential impact of such interventions on human disease. A separate QRA concluded that chemical decontamination agents such as chlorine could be powerful tools for decreasing *Salmonella* levels at the abattoir, these reductions were generally short-lived and negated by further processing steps [14]. As such, biocontrol agents such as phage which may retain some ability to infect *Salmonella* for some time after their application could produce a more sustained reduction in contamination than chemical agents. There is some experimental evidence that phage treatment can lead to sustained reductions of *Salmonella* numbers on the surface of chicken skin over 48 h at 4 °C [44]. An additional benefit of this would be that refrigerating the carcasses at 4 °C following phage treatment would not permit the regrowth of BIMs of *Salmonella*, which may not be the case if the phage were applied at farm level in live animals. Whether these effects can be replicated under commercial conditions has yet to be determined, however.

**Fig. 2:**
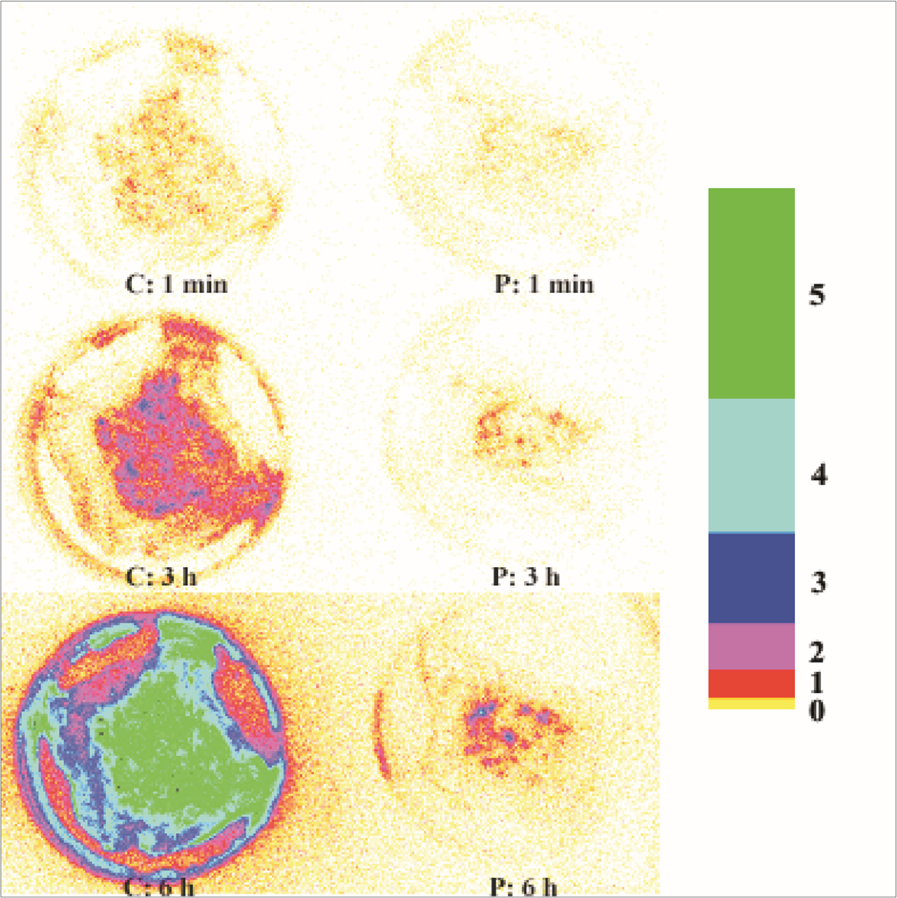
Time-lapse negative images of *Salmonella* growth on chicken skin following treatment with buffer (C) and phage (P) after 2 min, 3 h and 6 h, incubated at room temperature (~ 20 °C). A negative image of the logarithmic colour palette used by the Photek IFS32 software is presented on the right of the figure. Each colour represents the log_10_ number of photons collected at a given time point (1 min). The numbers on the right represent powers of ten (e.g. 2 = 10^2^ photons). All images were processed identically using Adobe® Photoshop 7.0 (Adobe Systems Inc., San Jose, USA) on a Viglen Genie XL280 PC running Microsoft Windows XP

The use of a high titer phage suspension increases the probability of ‘lysis from without’, especially for a pathogen such as *Salmonella* which is generally present in low numbers on carcasses [55]. The speed of the reduction in *Salmonella* numbers, as seen on the surface of chicken skin, suggests that at least some lysis from without rather than active replication on the skin or lysis during the counting procedure. This may also be considered as an additional step towards reduction of BIMs emergence. Moreover, the time-lapse photographs of bioluminescent *Salmonella* on the skin surface (Fig. 2) demonstrate that regrowth of *Salmonella* following phage treatment is significantly slowed when compared with the control, even when in a favourable food matrix and incubation temperature. This observation suggests that the benefits of phage treatment may extend beyond the processing plant, into the transport chain and possibly all the way to consumers’ kitchens, helping to reduce the risks of cross contamination during food preparation.

## Conclusions

This study has demonstrated that phages can be used to significantly reduce the surface contamination of chicken skin infected with *S.* Enteritidis and *S.* Typhimurium. The use of an autobioluminescent *Salmonella* strain supported the hypothesis that at least some of this reduction was due to lysis from without and occurred on the skin surface rather than in subsequent culture. The results of this study are promising, although further work needs to be done in order to optimise and adapt these phage treatments to an in-situ poultry industry setting.

## Supplementary information

**Additional file 1: Supplementary Table 1.** Lytic spectra of *Salmonella* bacteriophage Tφ11 determined on 35 *Salmonella* strains. Results were recorded as follows: +++, confluent lysis; ++, semiconfluent lysis; +, individual plaques, −, no lysis.

**Additional file 2: Supplementary Figure 1.** Transmission Electron Micrograph of phage Tφ11.

**Additional file 3: Supplementary Figure 2.** Transmission Electron Micrograph of phage Tφ11.

## Data Availability

The datasets used and/or analysed during the current study are available from the corresponding author upon reasonable request.

## Abbreviations

BIM: Bacteriophage insensitive mutants
CFU: Colony forming units
MPN: Most probable number
PFU: Plaque forming units
PT: Phage therapy
WHO: World Health Organization
